# Loss of *Fig4* in both Schwann cells and motor neurons contributes to CMT4J neuropathy

**DOI:** 10.1093/hmg/ddu451

**Published:** 2014-09-03

**Authors:** Ilaria Vaccari, Antonietta Carbone, Stefano Carlo Previtali, Yevgeniya A. Mironova, Valeria Alberizzi, Roberta Noseda, Cristina Rivellini, Francesca Bianchi, Ubaldo Del Carro, Maurizio D'Antonio, Guy M. Lenk, Lawrence Wrabetz, Roman J. Giger, Miriam H. Meisler, Alessandra Bolino

**Affiliations:** 1 Division of Neuroscience, INSPE-Institute of Experimental Neurology; 2 Department of Neurology and; 3 Division of Genetics and Cell Biology, San Raffaele Scientific Institute, Milan, Italy; 4 Department of Cell and Developmental Biology and; 5 Department of Human Genetics, University of Michigan, Ann Arbor, MI 48109, USA and; 6 Hunter James Kelly Research Institute, State University of New York, Buffalo, NY 14203, USA

## Abstract

Mutations of *FIG4* are responsible for Yunis-Varón syndrome, familial epilepsy with polymicrogyria, and Charcot-Marie-Tooth type 4J neuropathy (CMT4J). Although loss of the FIG4 phospholipid phosphatase consistently causes decreased PtdIns(3,5)*P*_2_ levels, cell-specific sensitivity to partial loss of FIG4 function may differentiate FIG4-associated disorders. CMT4J is an autosomal recessive neuropathy characterized by severe demyelination and axonal loss in human, with both motor and sensory involvement. However, it is unclear whether FIG4 has cell autonomous roles in both motor neurons and Schwann cells, and how loss of FIG4/PtdIns(3,5)*P*_2_-mediated functions contribute to the pathogenesis of CMT4J. Here, we report that mice with conditional inactivation of *Fig4* in motor neurons display neuronal and axonal degeneration. In contrast, conditional inactivation of *Fig4* in Schwann cells causes demyelination and defects in autophagy-mediated degradation. Moreover, Fig4-regulated endolysosomal trafficking in Schwann cells is essential for myelin biogenesis during development and for proper regeneration/remyelination after injury. Our data suggest that impaired endolysosomal trafficking in both motor neurons and Schwann cells contributes to CMT4J neuropathy.

## INTRODUCTION

In yeast and mammalian cells, the Fig4/FIG4 phospholipid phosphatase controls the generation and turnover of the PtdIns(3,5)*P*_2_ phosphoinositide, a regulator of membrane and protein trafficking at the level of the endosome–lysosome axis. Loss of Fig4/FIG4 causes a decrease of PtdIns(3,5)*P*_2_ levels and defects in multiple pathways in the endomembrane system. Typical cellular features associated with Fig4/FIG4 loss are the enlargement of late endosomes–lysosomes (LE/LY) and cytosolic vacuolization (1–3).

In human, recessive mutations in *FIG4* are responsible for the neurodegenerative Yunis-Varón syndrome, familial epilepsy with polymicrogyria, and Charcot-Marie-Tooth type 4J (CMT4J) neuropathy (3–10). Haploinsufficiency of *FIG4* may also be a risk factor for amyotrophic lateral sclerosis (ALS) (4).

Yunis-Varón syndrome is a severe disorder with autosomal recessive inheritance characterised by skeletal and structural brain abnormalities and facial dysmorphism (5). *FIG4* mutations identified in Yunis-Varón patients are nonsense or missense mutations that abolish FIG4 enzymatic activity, thus resulting in complete loss of FIG4 function (5,9). Recently, a homozygous missense mutation causing partial loss of FIG4 function was demonstrated to co-segregate with polymicrogyria, psychiatric manifestations and epilepsy in a consanguineous Moroccan family, thus suggesting a role for FIG4 in the regulation of cortical brain development (10). ALS is a severe neurological disorder characterized by selective neurodegeneration of lower and upper motor neurons. ALS patients carrying mutations in *FIG4* are heterozygous for a null allele (deletions or splice site mutations leading to frameshift) or for missense mutations which alter FIG4 enzymatic activity (4). Patients with CMT4J neuropathy display a variable degree of severity. Early onset CMT4J shows asymmetrical motor and sensory neuropathy, which is usually rapid in progression. Late onset CMT4J displays a prevalent motor and asymmetric neuropathy, which is a typical feature of lower motor neuron disease rather than of CMT neuropathy (6). However, in both early and late onset CMT4J, the reduction of nerve conduction velocity (NCV) and the presence of onion bulbs in nerve biopsy suggest a demyelinating type of CMT, thus being classified in the CMT4 subclass (6–8). CMT4J patients are compound heterozygous for one missense mutation and one loss-of-function mutation. The I41T allele is the most frequent CMT4J missense mutation, and partially affects FIG4 enzymatic activity by destabilizing the protein (3,11).

Overall, these disorders indicate that, despite the ubiquitous expression, loss of FIG4 affects specific cell types with distinct pathogenetic mechanisms. This cell-specific effect might be due to the impact of the different mutations on the FIG4 enzymatic activity/stability and/or to the impairment of cell-specific functions within the endolysosome axis. These aspects have been only partially elucidated using the *Fig4*-null mouse models generated so far. For example, while available mouse models clearly indicate a predominant role for Fig4 in neurons, the onion bulbs and active demyelination/remyelination observed in CMT4J patient biopsies would be consistent with a cell autonomous role for FIG4 in Schwann cells as well (6–8).

Here, we report the generation and characterization of mouse mutants with conditional inactivation of *Fig4* in either motor neurons or Schwann cells, two cell types affected in the CMT4J neuropathy. We found that *Fig4* loss in motor neurons causes neuronal and axonal degeneration, whereas the *Fig4*-Schwann cell conditional mutant displays a general trafficking impairment, leading to a defect in autophagy-mediated degradation and demyelination. We also exploited the *Fig4*-Schwann cell conditional mutant to investigate whether trafficking through the endolysosome axis contributes to myelin biogenesis during development and to regeneration/remyelination. Our *in vitro* and *in vivo* data suggest that altered LE/LY homeostasis in Schwann cells impairs both active myelination and nerve regeneration.

## RESULTS

### Loss of *Fig4* in motor neurons *in vivo* leads to neuronal and axonal degeneration

**Figure 1.**
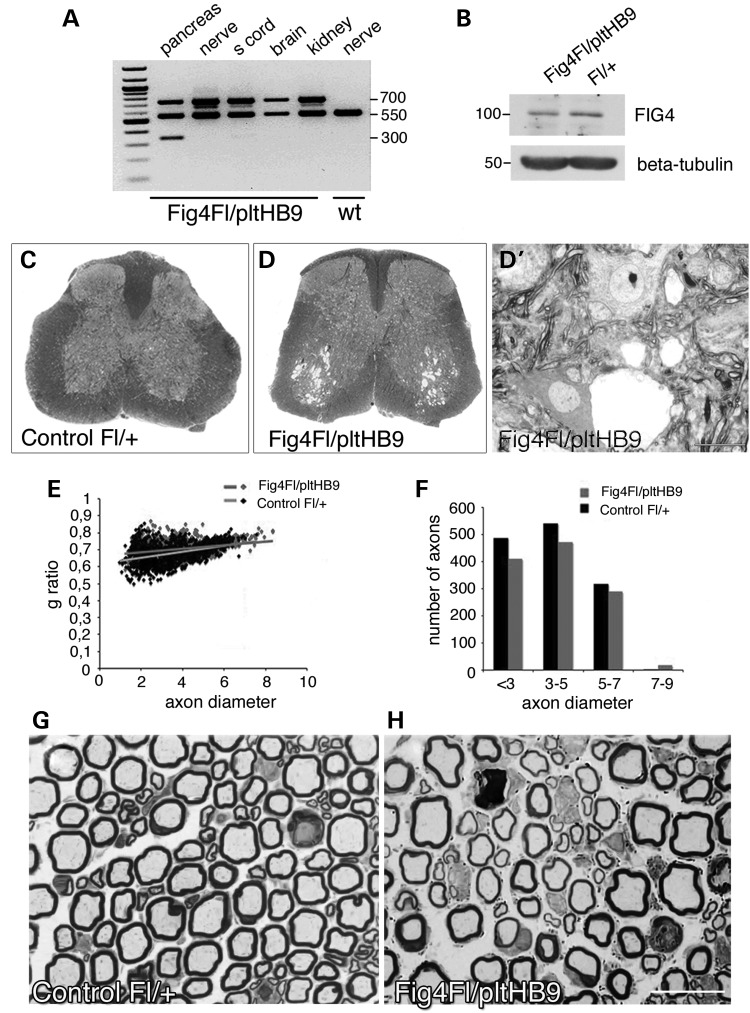
Conditional ablation of *Fig4* specifically in motor neurons. (**A**) PCR analysis of genomic DNA from *Fig4^Floxed/plt^*, *HB9*-Cre mice and controls. A 300-bp recombination band was detected in the pancreas where *HB9* is highly expressed. A faint band is also present in spinal cord, which contains other cells in addition to motor neurons where recombination occurs. (**B**) Western blot analysis demonstrated decreased Fig4 expression in lysates from motor roots and ventral horn of mutant mice at P30. (**C** and **D′**) Toluidine blue staining of spinal cords from *Fig4^Floxed/plt^*, *HB9*-Cre mice at P30 shows vacuolization in the ventral horns where motor neurons are located (L4–L5). (**E** and **F**) G-ratio analysis of quadriceps nerves at P30 indicates reduction of myelin thickness in *Fig4^Floxed/plt^*, *HB9*-Cre mouse nerves. The total number of fibres and axon diameter distribution are normal in *Fig4^Floxed/plt^*, *HB9-*Cre P30 quadriceps nerves. (**G** and **H**) Semithin section analysis of quadriceps nerves at P90 shows hypomyelination and reduced density of fibres in mutant *Fig4^Floxed/plt^*, *HB9-*Cre nerves. Bar in (D′) is 10 µm and in (H) is 10 µm.

CMT4J patients initially display a prevalent motor and asymmetric neuropathy, which is a typical feature of a lower motor neuron disease rather than of demyelinating CMT neuropathies (6,7). This observation suggests that lower motor neurons are vulnerable to loss of Fig4. Mutants investigated thus far include the *Fig4^plt/plt^* mouse (a spontaneous mutant with global *Fig4* loss), the *Fig4^Floxed/Floxed^*, *Syn*-Cre conditional mutant lacking *Fig4* specifically in neurons and the *Fig4^plt/plt^*, *NSE*-*Fig4*(tg) mouse overexpressing *Fig4* specifically in neurons under the control of the neuron-specific promoter *NSE*. Analysis of all of these mutants demonstrates that *Fig4* plays an important role in neurons (1,3,12). However, in the *Fig4^plt/plt^* mouse, spinal motor neurons were among the last neurons to exhibit vacuolization, being largely preserved at P21 but filled with vacuoles at 6 weeks of age (3,13). The lethality of the *Fig4^plt/plt^* mice ∼6 weeks of age did not permit further evaluation of the *Fig4* loss-of-function phenotype in motor neurons. Thus, for a more specific assessment of *Fig4* in motor neurons and their peripheral projections, we generated *Fig4^Floxed/plt^, HB9-*Cre mice, in which the *HB9-*Cre transgene produces somatic recombination at embryonic day 9.5 (E9.5) in motor neurons and in the pancreas (14–17). To achieve maximal efficiency of *HB9*-Cre-mediated recombination, we generated compound heterozygous mice carrying one null allele (*Fig4^plt^*, global Fig4 deficiency) and one Floxed allele at the *Fig4* locus. Heterozygous *Fig4^plt/+^* mice and homozygous *Fig4^Flox/Flox^* mice are normal in survival and morphology, as previously reported (3,12,18). PCR analysis of genomic DNA demonstrated *HB9-*Cre-mediated excision of *Fig4* in the pancreas and partial excision in the spinal cord, which also contains non-neuronal cells (Fig. 1A). Western blot analysis of lysates from ventral horns and motor roots of spinal cords also showed decreased Fig4 expression in *Fig4^Floxed/plt^*, *HB9-*Cre mice (Fig. 1B). *Fig4^Floxed/plt^*, *HB9-*Cre spinal cords at P30 and P90 display extensive cell vacuolization in the ventral horn where motor neurons are located (Fig. 1C and D′ and data not shown). Moreover, quadriceps nerves from *Fig4*^*Floxed/plt*^, *HB9-*Cre mice displayed mild hypomyelination with increased g-ratio (the ratio between axon diameter and fibre diameter) at P30 (Fig. 1E and F; g-ratio: *Fig4^Floxed/plt^*, *HB9-*Cre 0.71 ± 0.0004, 1189 fibres; controls *Fig4^Fl/+^* 0.68 ± 0.003, 1350 fibres; *n* = 4, *P* = 0.0057). This was also observed at P90, when signs of axonal degeneration and fibre loss were evident (Fig. 1G and H; number of fibres at P90: *Fig4^Floxed/plt^, HB9-*Cre 477 ± 11.5 and controls *Fig4^Fl/+^* 536 ± 7.9, *n* = 3, *P* = 0.01). At 6 and 12 months of age, these *Fig4^Floxed/plt^*, *HB9*-Cre mice were viable and clinically indistinguishable from control mice, and did not display tremor or gross behavioural impairment.

Consistent with the observations with *HB9*-Cre, we also observed motor neuron vacuolization in spinal cords of *Fig4^Floxed/plt^*, *Olig2*-Cre mice (Supplementary Material, Fig. S1), where the *Olig2* promoter drives Cre expression starting at E10.5 in *Shh* (Sonic Hedgehog) responsive domains of the neural tube that give rise to motor neurons as well as oligodendrocyte precursor cells (OPCs) (19,20).

These results demonstrate that *Fig4* has a cell autonomous role in motor neurons since *Fig4^Floxed/plt^*, *HB9-*Cre mutants show neuronal and axonal degeneration, leading to mild hypomyelination likely as a secondary consequence of altered axo-glial communication. Moreover, the fact that demyelination is not observed in the nerves of the motor neuron conditional *Fig4^Floxed/plt^*, *HB9-*Cre mouse supports the hypothesis that loss of *Fig4* in Schwann cells may contribute to demyelinating CMT4J.

### Loss of *Fig4* in Schwann cells *in vivo* causes a progressive demyelinating neuropathy

To directly assess a Schwann cell autonomous role of *Fig4*, we generated both *Fig4^Floxed/Floxed^*, *P0*-Cre and *Fig4^Floxed/plt^*, *P0*-Cre mice, in both of which *Fig4* is specifically ablated in Schwann cells starting from E13.5 (17,21–23).

**Figure 2.**
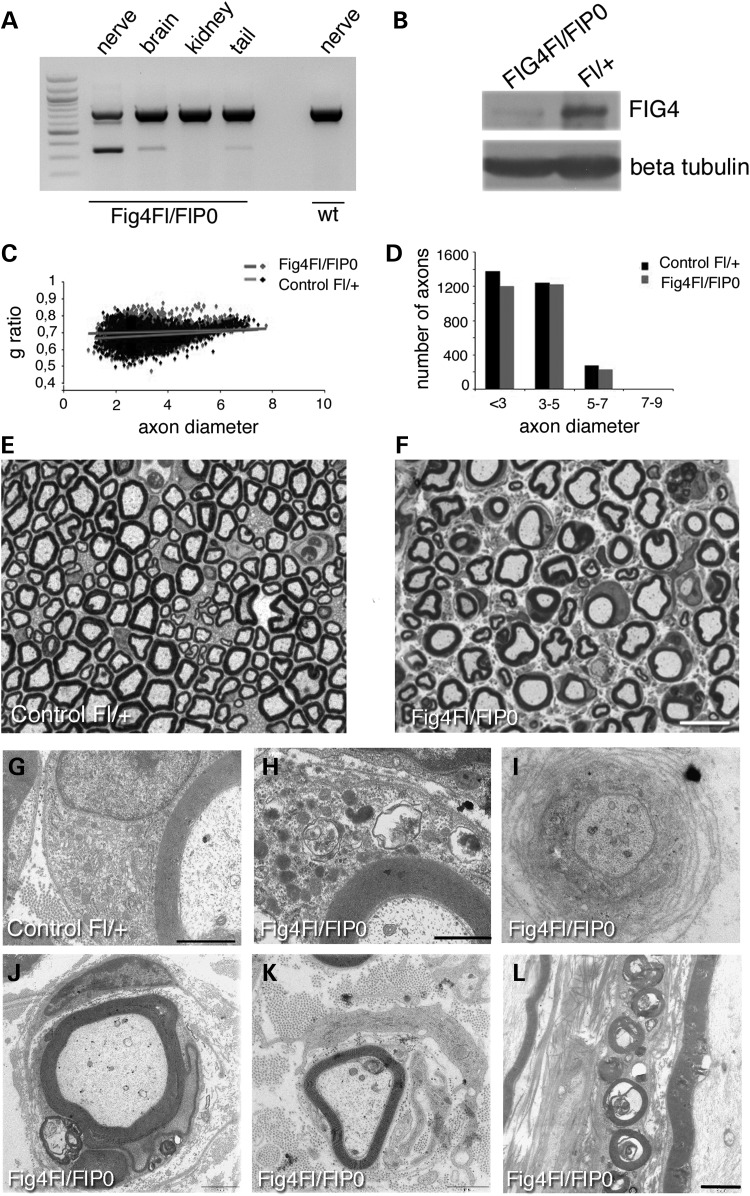
Schwann cell conditional ablation of *Fig4* causes developmental hypomyelination and demyelination in the *Fig4^Floxed/Floxed^*, *P0*-Cre mouse. (**A**) PCR analysis on genomic DNA from *Fig4^Floxed/Floxed^*, *P0*-Cre mice and controls. A 300-bp recombination band was detected in the nerve of mutants but not in wild type. The faint 300-bp band in the brain probably indicates recombination in cranial nerves (21,22) and in Schwann cells in the tail nerve. (**B**) Western blot analysis shows reduction of Fig4 in mutant sciatic nerves at P30. (**C** and **D**) G-ratio analysis of sciatic nerves at P60 indicates decreased myelin thickness and hypomyelination in *Fig4^Floxed/Floxed^*, *P0*-Cre mouse nerves. (**E** and **F**) Semithin section analysis of sciatic nerves at 4 months showed the presence of vesicles/myelin debris in the Schwann cell cytoplasm of mutant myelinated fibres, observed in ultrastructural analysis at P30 in (**H**), when compared with a control at the same age (**G**). (**I** and **L**) Ultrastructural analysis of sciatic nerves at 4 months showed the presence of demyelination such as onion bulbs (I**–K**) and myelin debris (L). Bar in (E and F) is 10 µm, in (G) is 0.7 µm and in (H) is 0.88 µm. Bar in (L) is 0.5 μm for (I) and 2 μm for (J–L).

**Figure 3.**
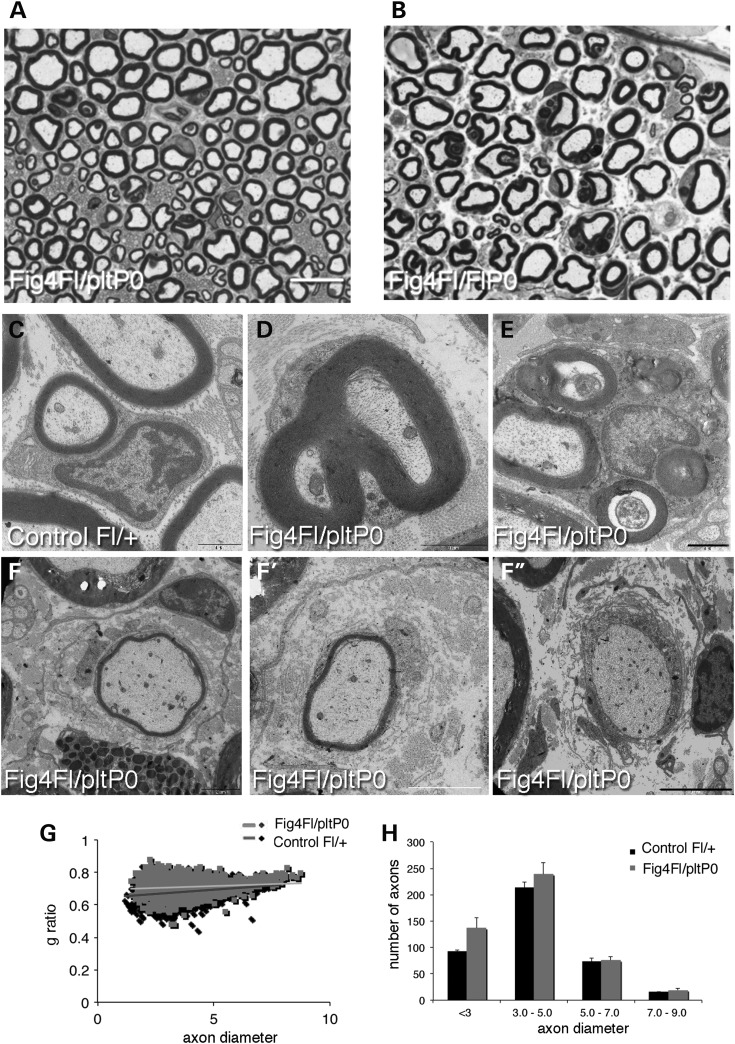
Schwann cell conditional ablation of *Fig4* in the *Fig4^Floxed/plt^*, *P0*-Cre mouse resulted in a milder phenotype. (**A** and **B**) Semithin section analysis of sciatic nerves of *Fig4^Floxed/plt^*, *P0*-Cre when compared with *Fig4^Floxed/Floxed^*, *P0*-Cre at 4 months. The percentage of fibres carrying vesicles/myelin debris in the Schwann cell cytoplasm is higher in *Fig4^Floxed/Floxed^*, *P0*-Cre mice. Myelinated fibres from *Fig4^Floxed/plt^*, *P0*-Cre sciatic nerves at 8 months showing demyelinating features such as redundant myelin (**D**), myelin degeneration (**E**), and onion bulbs (**F**–**F′′**). *Fig4^Floxed/plt^*, *P0*-Cre sciatic nerves analysed at P30 (**G** and **H**) have increased g-ratio values indicating developmental hypomyelination. Bar in (A) is 10 µm for (A and B); bar in (F**′′**) is 1.7 µm for (**C**); 1 µm for (D); 1.7 µm for (E); 3 µm for (F); 2.4 µm in (F′) and 5 µm for (F′′).

**Figure 4.**
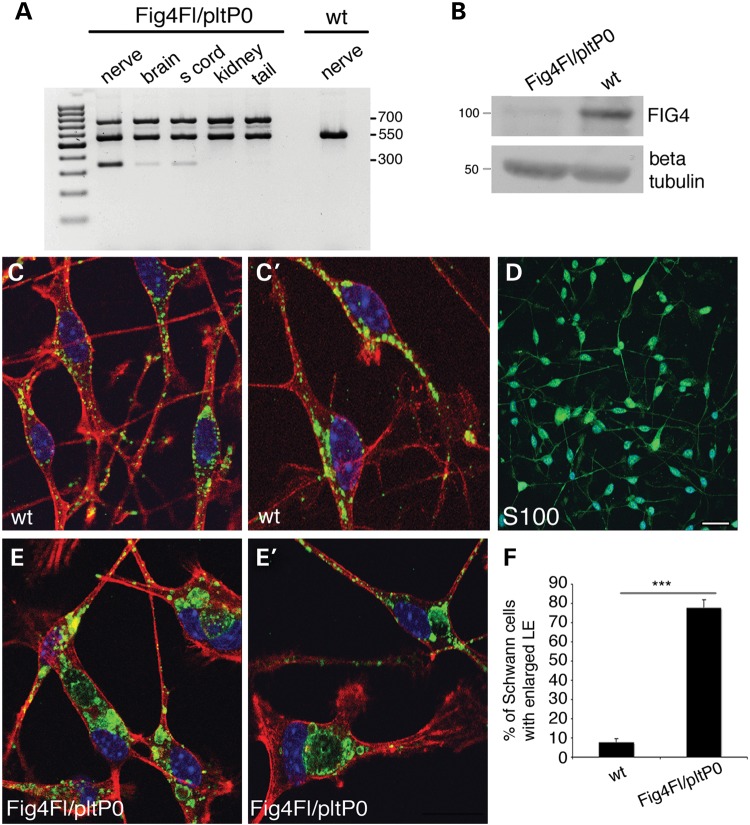
*P0*-Cre-mediated recombination efficiency in the *Fig4^Floxed/plt^*, *P0*-Cre model. (**A**) PCR analysis on genomic DNA from *Fig4^Floxed/plt^*, *P0*-Cre mice and controls. A 350-bp recombination band was detected in the nerve of mutants but not in wild type. The 350-bp faint band in the brain and spinal cord indicates recombination in cranial and spinal nerves of brain and spinal cord, respectively. (**B**) Western blot analysis of nerve homogenates at P30 indicates decreased Fig4 expression in sciatic nerves of *Fig4^Floxed/plt^*, *P0*-Cre mice. (**C–F**) Purified Schwann cells from *Fig4^Floxed/plt^*, *P0*-Cre mouse nerves at P3 indicate the presence of enlarged LE/LY-LAMP1 positive (green in C, **C**′, E, **E′**; red is phalloidin; blue is DAPI) in mutant cells, a feature of Fig4 loss and PtdIns(3,5)*P*_2_ decrease. (D) S100 staining marks Schwann cells in the culture. Bar in (D) is 20 µm for (C, C′, E, E′) and 50 µm for (D).

To demonstrate *P0*-Cre-mediated ablation of *Fig4* in Schwann cells of *Fig4^Floxed/Floxed^*, *P0*-Cre mouse nerves, we performed PCR analysis of genomic DNA from different tissues. Recombination of the Floxed allele in sciatic nerve and brain was detected by generation of a 300 bp PCR product (Fig. 2A). Western blot analysis confirmed reduced Fig4 expression in *Fig4^Floxed/Floxed^*, *P0*-Cre mouse sciatic nerve (Fig. 2B). Morphological analysis of sciatic nerve revealed an accumulation of organelles and of lipidic material/vesicles in the cytoplasm of myelinating Schwann cells in *Fig4^Floxed/Floxed^*, *P0*-Cre nerve fibres at P30, P60 and 4 months of age, suggesting a general trafficking impairment (Fig. 2F and H and Fig. 3B). The assembly of myelin membrane during myelination depends on polarized trafficking of lipids and proteins through endocytic routes (24,25). Consistent with this, *Fig4^Floxed/Floxed^*, *P0*-Cre mutant sciatic nerves also displayed reduced myelin thickness and increased g-ratio values at P60 (Fig. 2C and D), suggesting that the regulation of endocytic trafficking through the endolysosomal axis is essential for myelination (g-ratio: *Fig4^Floxed/Floxed^, P0*-Cre 0.71 ± 0.005, 2655 fibres; controls *Fig4^Floxed/+^* 0.68 ± 0.005, 2832 fibres, *n* = 4, *P* = 0.009). In older *Fig4^Floxed/Floxed^*, *P0*-Cre nerves at 4 months we observed progression of the phenotype with demyelinating features including onion bulbs (1% of the total number of fibres in sciatic nerves—onion bulbs have never been observed in control nerves), myelin degeneration (Fig. 2I–L), and a further reduction in the myelin thickness when compared with developmental stages (g-ratio: *Fig4^Floxed/Floxed^*, *P0*-Cre 0.72 ± 0.001, 880 fibres and controls *Fig4^Floxed/+^* 0.68 ± 0.001, 1416 fibres, *n* = 3; *P* = 6 28178E−06). Finally, at 4 months demyelination was also associated with fibre loss (total number of fibres: *Fig4^Floxed/Floxed^*, *P0*-Cre 2368 ± 56.45 fibres and controls *Fig4^Floxed/+^* 2978 ± 152.9 fibres, *n* = 3; *P* = 0.0191; Figs. 2F and 3B).

Morphological analysis of sciatic nerve from *Fig4^Floxed/plt^*, *P0*-Cre mice also revealed abnormal cytosolic enlarged vacuoles, accumulation of vesicles, organelles and myelin debris (Fig. 3A and B). *Fig4^Floxed/plt^*, *P0*-Cre mouse nerves also exhibit mild hypomyelination with increased g-ratio values observed at P30 (Fig. 3G and H) that is slightly worsened at 8 months of age, together with occasional onion bulbs (Fig. 3F–F′′; g-ratio at P30: *Fig4^Floxed/plt^*, *P0*-Cre 0.70 ± 0.003, 2161 fibres and controls *Fig4^Floxed/+^* 0.68 ± 0.003, 2488 fibres, *n* = 4, *P* = 0.009 and controls *Fig4^Floxed/plt^* 0.68 ± 0.002, *n* = 2300 fibres, *P* = 0.001; g-ratio at 8 months: *Fig4^Floxed/plt^*, *P0*-Cre 0.71 ± 0.001, 1640 fibres and controls *Fig4^Floxed/+^* 0.68 ± 0.001, 2023 fibres; *n* = 5, *P* = 1.12833E−06). Note that *Fig4^plt/+^* nerves have normal g-ratio values at both P30 and 4.5 months (data not shown) and as previously reported (18). Fibre loss was not observed in *Fig4^Floxed/plt^*, *P0*-Cre sciatic nerves at 8 months (total number of fibres: *Fig4^Floxed/plt^*, *P0*-Cre 2279 ± 72.5 and controls *Fig4^Floxed/+^* 2229 ± 12.5; *n* = 4, *P* = 0.52). Neurophysiological analysis was consistent with a demyelinating neuropathy with reduced NCV (*Fig4^Floxed/plt^*, *P0*-Cre 29.07 ± 0.579 and controls *Fig4^Floxed/+^* 40.84 ± 0.773, *P* = 1.91553E−06) and increased F-wave latency (*Fig4^Floxed/plt^*, *P0*-Cre 6.25 ± 0.2903 and controls *Fig4^Floxed/+^* 4.957 ± 0.105, *n* = 8, *P* = 0.0031). Cre-mediated recombination was demonstrated in *Fig4^Floxed/plt^*, *P0*-Cre by PCR analysis of genomic DNA and by western blot analysis of nerve lysates, which clearly demonstrated a strong reduction of Fig4 expression (Fig. 4A and B). To further assess the efficiency of *P0*-Cre-mediated recombination, we cultured primary mouse Schwann cells established from *Fig4^Floxed/plt^, P0*-Cre and control nerves at P3. Immunohistochemistry for LAMP1, which is a marker of LE/LY, demonstrated that 70% of mutant Schwann cells carried enlarged LE/LY (Fig. 4C–F), thus confirming loss of Fig4/PtdIns(3,5)*P*_2_-mediated control of LE/LY homeostasis in these cells.

We attribute the somewhat milder phenotype of *Fig4^Floxed/plt^*, *P0*-Cre mice, compared with *Fig4^Floxed/Floxed^*, *P0*-Cre mice, to the greater contribution of genetic background from strain C57BL/6J, which is known to exacerbate *Fig4*-null associated features (1,3,26). The *Fig4^plt^* allele was maintained in a mixed strain background whereas the *Fig4^Floxed/Floxed^, P0*-Cre genotype was enriched in the C57BL/6J strain (3,12).

Overall, these findings demonstrate that Schwann cells are vulnerable to loss of *Fig4* and that loss of *Fig4* in Schwann cells reproduces demyelinating features of human CMT4J neuropathy.

### Loss of *Fig4* in Schwann cells *in vivo* impairs nerve regeneration

**Figure 5.**
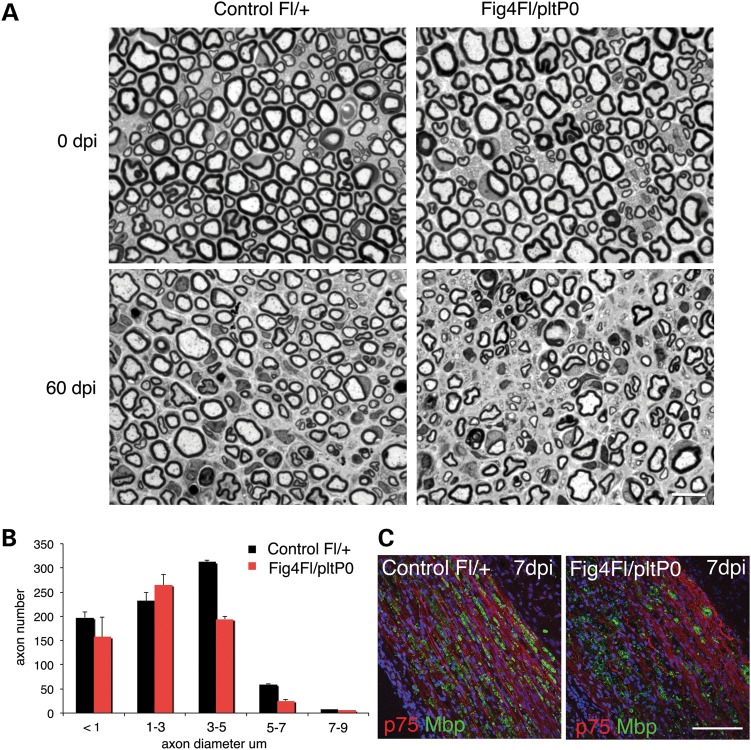
Nerve regeneration is impaired in the *Fig4^Floxed/plt^*, *P0*-Cre conditional knock out. (**A**) Semithin section analysis of sciatic nerves from controls (left) and *Fig4^Floxed/plt^*, *P0*-Cre mutant nerves (right panels) analysed at 60 dpi (days post injury-crush performed at 2.5 months) indicates that regeneration is impaired in this mutant. Fibre loss is also observed in mutant nerves after injury. (**B**) The distribution of the number of axons per diameter indicates that fibres in the range of 3–5 µm (*P* = 0.006) and 5–7 µm of diameter (*P* = 0.001) were significantly decreased in the *Fig4^Floxed/plt^*, *P0*-Cre nerves after crush. (**C**) Staining of *Fig4^Floxed/plt^*, *P0*-Cre nerves at 7 dpi using anti-MBP antibody (green) which marks myelin and anti-p75 (red), which labels trans-differentiating Schwann cells. Bar in (A) is 20 µm; in (C) is 100 µm.

Loss of *Fig4* in Schwann cells was shown above to be associated with demyelination and progressive axonal loss. Since impaired regeneration may also contribute to axonal loss in CMT neuropathies, we investigated whether impaired trafficking through the endolysosome axis in *Fig4*-null Schwann cells could affect remyelination and nerve regeneration. To this aim, we exploited the milder *Fig4^Floxed/plt^ P0*-Cre mouse model to prevent developmental degeneration that might interfere with nerve regeneration after injury. Sciatic nerves from *Fig4^Floxed/plt^*, *P0*-Cre mice and controls were crushed at 2 months of age, a time point at which myelination and axonal integrity are largely normal, and morphological analysis was performed 60 days after injury (Fig. 5A). As expected, regeneration of axons and myelin was nearly complete in control nerves. In contrast, in *Fig4^Floxed/plt^*, *P0*-Cre crushed nerves, we observed a reduced number of medium and large calibre fibres and total fibres, and the presence of myelin debris (Fig. 5A and B; *Fig4^Floxed/plt^*, *P0*-Cre 2537 fibres when compared with control *Fig4^Floxed/+^* 1782 fibres, *n* = 4 and *P* = 0.002). Heterozygous *Fig4^plt^*^/+^ crushed nerves were indistinguishable from controls (18) (and data not shown).

Since specific loss of *Fig4* in Schwann cells appears to cause an endolysosomal trafficking impairment, we asked whether the altered regeneration of myelinated fibres could be caused by delayed clearance of myelin debris by Schwann cells. Immunohistochemistry of nerves at 7 days after injury demonstrated that MBP protein level was similar between non-regenerating *Fig4^Floxed/plt^*, *P0*-Cre nerves and controls, indicating normal myelin clearance (Fig. 5C). This finding was also supported by morphological analysis of semithin sections (data not shown).

In conclusion, loss of *Fig4* in Schwann cells impairs regeneration of myelinated fibres suggesting that *Fig4* and PtdIns(3,5)*P*_2_ homeostasis are necessary for efficient nerve regeneration.

### Loss of *Fig4* in Schwann cells impairs endolysosomal homeostasis resulting in hypomyelination *in vitro*

**Figure 6.**
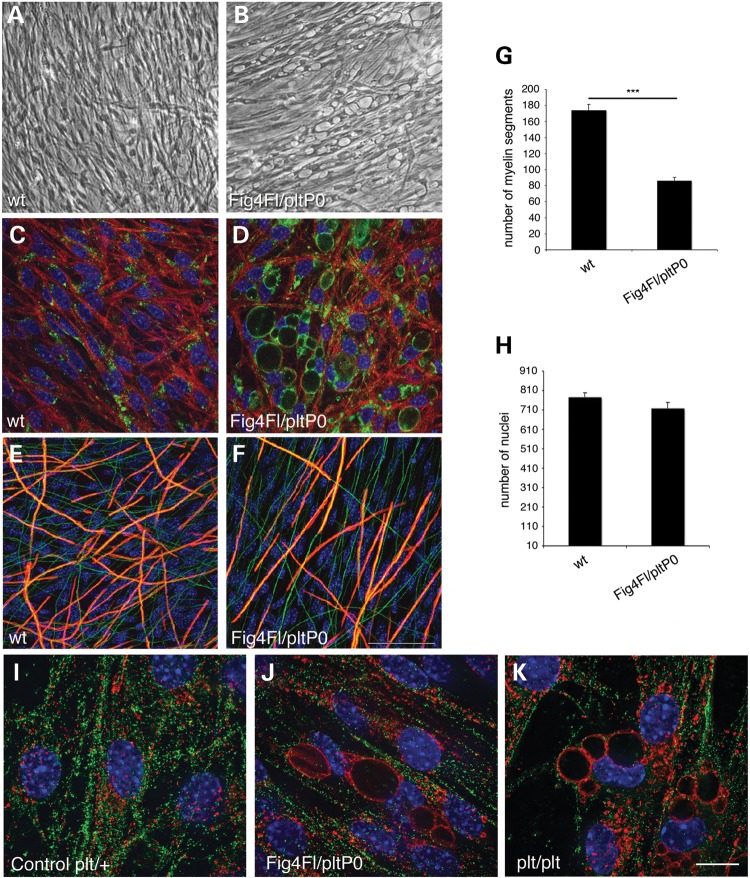
Schwann cell/DRG neuron co-cultures from *Fig4^Floxed/plt^*, *P0*-Cre mice are hypomyelinated. (**A** and **B**) Bright field images showing vacuolization of Schwann cells in explants established from *Fig4^Floxed/plt^*, *P0*-Cre mouse embryos, which corresponds to LE/LY compartment LAMP1 positive (LAMP1 green, red phalloidin and blue DAPI) in (**C** and **D**). (**E**–**H**) Vacuolated Schwann cells produced less myelin segments after 7 days of ascorbic acid treatment when compared with control cultures (red is MBP, green is neurofilament and blue is DAPI), *n* = 6–8 explants per genotype/three different experiments. (**I** and **J**) Myelin protein zero, P0, does not co-localize with LAMP1 in control and in *Fig4^Floxed/plt^*, *P0*-Cre Schwann cells (green is P0, red is LAMP1 and blue is DAPI), *n* = 4 explants per time point of ascorbic acid treatment, per genotype. Two different anti-P0 antibodies were used. Bar in (F) is 150 µm for (A and B) and 50 µm for (C–F); bar in (**K**) is 13 µm for (I–K).

We showed above that loss of *Fig4* specifically in Schwann cells causes a general trafficking defect, an impairment of active myelination during development, and demyelination. To determine how loss of *Fig4* in Schwann cells and the consequent impairment of PtdIns(3,5)*P*_2_-mediated trafficking affect myelination during development, we established myelin-forming Schwann cell/DRG neuron co-culture explants from *Fig4^Floxed/plt^*, *P0*-Cre mice, in which *P0*-Cre-mediated recombination should be more efficient *in vitro* due to the presence of a single Floxed allele. Under myelinating conditions, following ascorbic acid treatment, most Schwann cells displayed enlarged LAMP1-positive LE/LY (Fig. 6A–D). Although *Fig4^Floxed/plt^*, *P0*-Cre explants were able to myelinate, the number of MBP-positive segments was significantly reduced when compared with control cultures (Fig. 6E–G). The Schwann cell number was similar between mutant and control (Fig. 6H). This finding is consistent with the observed hypomyelination in mutant mice and confirms that reduced PtdIns(3,5)*P*_2_ in Schwann cells results in impaired myelination. Note that myelin production was equivalent in *Fig4^+/+^*, *Fig4^plt/+^* and *Fig4^plt/Floxed^* cultures, consistent with the normal *in vivo* phenotype of *Fig4^plt/+^* mice (data not shown).

To explore the molecular basis of hypomyelination, we determined whether loss of *Fig4* in Schwann cells and the enlargement of LE/LY results in impaired exocytosis of myelin proteins, impaired myelin protein degradation or more general defects in the endomembrane system during active myelination. It has been recently suggested that P0 co-localizes with LAMP1-positive compartments at early stages of postnatal nerve development (27). Myelin protein zero (P0) may be stored in LE/LY compartments prior to exocytosis during myelin assembly, as proposed for PLP in oligodendrocytes in the CNS (28), or during remyelination after damage. To explore this possibility, we evaluated retention of P0 in the enlarged LE/LY compartment of *Fig4*-null Schwann cells. Explants were stained with anti-P0 and anti-LAMP1 antibodies after ascorbic acid treatment, at 1–6 days of ascorbic acid treatment. We did not observe co-localization of P0 and LAMP1 in mutant or control cultures (Fig. 6I–K).

We then investigated whether altered protein degradation or turnover contributes hypomyelination in mutant cells. To this aim, we determined levels of autophagic markers and ubiquitinated substrates by western blot analysis. We did not observe an elevation of LC3II/I, p62 or ubiquitinated substrates in *Fig4^Floxed/plt^, P0*-Cre explants when compared with controls after 7 days of ascorbic acid treatment (Supplementary Material, Fig. S2A–C), suggesting that hypomyelination is not a consequence of impaired protein turnover.

**Figure 7.**
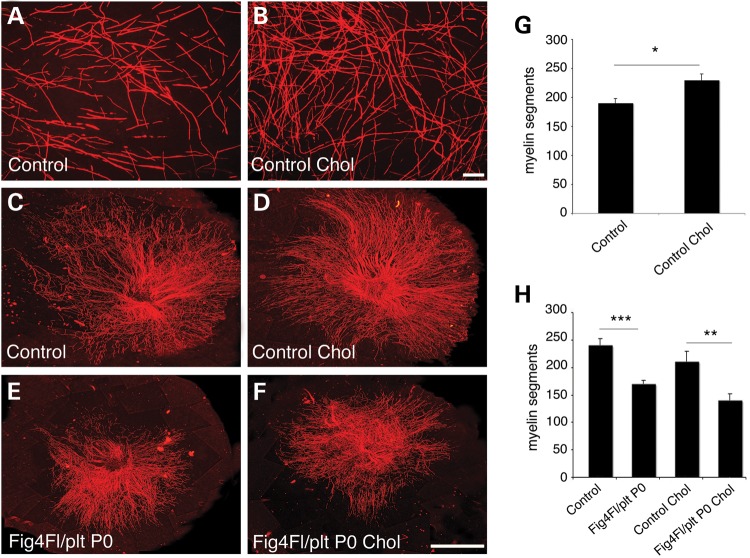
Cholesterol supplementation does not rescue *Fig4^Floxed/plt^*, *P0*-Cre hypomyelination *in vitro*. (**A** and **B**) Cholesterol supplementation accelerates myelination of control explants after 7 days of ascorbic acid treatment, quantified in (**G**), *P* = 0.03305, *n* = 6. (**C**–**F**) Cholesterol supplementation does not rescue hypomyelination in *Fig4^Floxed/plt^*, *P0*-Cre cultures following 7 days of ascorbic acid treatment. (**H**) Cholesterol supplementation does not rescue hypomyelination also after 15 days of ascorbic acid treatment, as myelin segments in mutant cultures were decreased when compared with controls without cholesterol supplementation (*P* = 0.00071) and with cholesterol supplementation (*P* = 0.00868), *n* = 6. (H) Note that, after 15 days of cholesterol supplementation, control cholesterol-treated and untreated cultures produced the same amount of myelin, as previously reported (29). Red is MBP, myelin basic protein. Bar in (B) and (F) is 50 µm. Control genotypes are *Fig4^Floxed/plt^* and *Fig4^Floxed/Floxed^*.

Cholesterol is known to facilitate the exit of P0 from the Golgi compartment and delivery to the plasma membrane, and addition of cholesterol to myelin-forming cultures accelerates myelination (29). We reasoned that if hypomyelination in *Fig4*^Floxed/*plt*^, *P0*-Cre mutant cultures was the consequence of impaired membrane trafficking in the endomembrane system, cholesterol delivery to the plasma membrane would itself be impaired and therefore addition of cholesterol to the culture medium would not rescue hypomyelination. Control cultures treated with cholesterol displayed enhanced myelination after 7 days of treatment, but not at 13 days, in agreement with previous reports (29) (Fig. 7A–D, and quantification in G). Consistent with our hypothesis, treatment of mutant explants with ascorbic acid in the presence of 20 µg/μl cholesterol did not rescue hypomyelination after 7 or 14 days of treatment (Fig. 7E and F, and quantification in H). This finding suggests that in mutant cells cholesterol trafficking and/or P0 delivery and assembly to the plasma membrane is not as efficient as in controls due to defects in the endolysosomal trafficking.

### Altered endolysosomal trafficking results in a block of autophagic progression and demyelination

**Figure 8.**
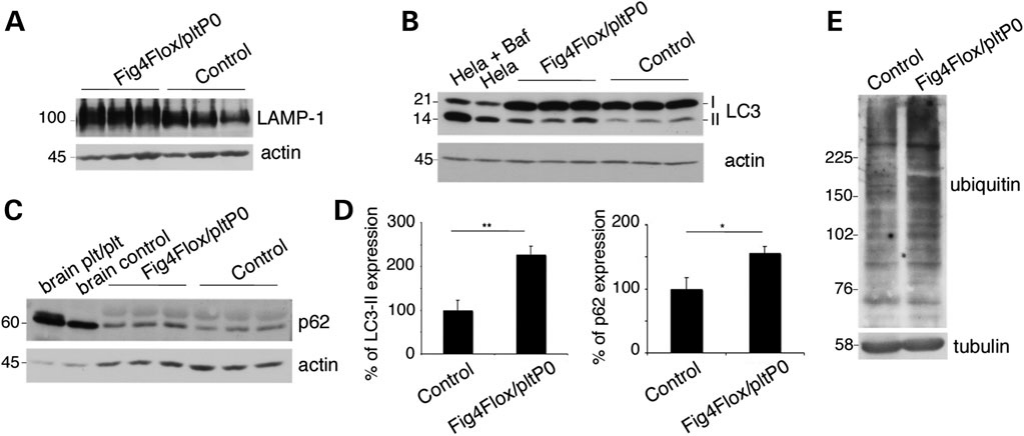
Block of autophagic progression in *Fig4^Floxed/plt^*, *P0*-Cre nerves. (**A**–**D**) LAMP1, LC3II/I and p62 levels are increased in P30 sciatic nerves of *Fig4^Floxed/plt^*, *P0*-Cre mice. An elevation of p62 indicates a block in autophagic progression in Schwann cells. In (B), lysates from HeLa cells starved and starved-treated using Bafalomycin, which blocks lysosomal-mediated degradation, were used as controls of LC3 II/I elevation. In (C), brain lysates from *Fig4^plt/plt^* and controls were used as a positive control of p62 increase, as already reported (1,33). (**E**) Lysates of sciatic nerves from *Fig4^Floxed/plt^*, *P0*-Cre mice and controls at P90 were stained using an anti-polyubiquitin antibody. Control genotypes are *Fig4^Floxed/plt^* and *Fig4^Floxed/Floxed^*.

We then asked how loss of *Fig4* in Schwann cells and the consequent impairment of PtdIns(3,5)*P*_2_-mediated trafficking can lead to the observed demyelination in the nerve. *Fig4* loss and/or reduced activity of the PIKfyve kinase complex that generates PtdIns(3,5)*P*_2_ from PtdIns3*P*, leads to decreased PtdIns(3,5)*P*_2_ levels and enlargement of the LE/LY compartment (2,30–32). A block in autophagic progression has been reported in astrocytes and, to a lesser extent, in neurons of *Fig4^plt/plt^* mice (1,3,33). This defect may result from reduced fusion between enlarged LE/LY and autophagosomes to form autophagolysosomes (33). We thus asked whether there is a block of autophagy in *Fig4*-null Schwann cells. Western blot analysis of sciatic nerve lysates from *Fig4*^*Floxed*/*plt*^, *P0*-Cre mice at P30 revealed that the levels of LAMP1, LC3II and p62 proteins were significantly increased, consistent with a block in autophagic flux (Fig. 8A–D). Accumulation of ubiquitinated proteins is characteristic of impaired autophagy (34). We analysed levels of ubiquitinated proteins in P90 *Fig4^Floxed/plt^*, *P0*-Cre nerve lysates and found an increase of polyubiquitinated proteins in mutant nerves when compared with control (Fig. 8E).

The data demonstrate that *Fig4* loss in Schwann cells causes both enlarged LE/LY and a block of autophagic flux, which may lead to demyelination.

## DISCUSSION

### The CMT4J neuropathy: cell autonomy of *Fig4* in motor neurons and Schwann cells

Loss of human *FIG4* causes a spectrum of inherited conditions suggesting that more than one cell type is vulnerable to impairment of FIG4/PtdIns(3,5)*P*_2_-mediated functions (3–7,9,10). This cell-specific sensitivity could be related to the impact of the different mutations on FIG4 enzymatic activity and protein stability, as well as to the impairment of different cellular functions in different cell types.

The CMT4J neuropathy is classified as CMT type 4 because of the autosomal recessive inheritance and of demyelinating features such as the reduction of NCV and the presence of onion bulbs in the nerve biopsies (6–8). However, late onset CMT4J patients initially display a predominantly motor and asymmetric neuropathy, suggesting a lower motor neuron involvement in the pathogenesis of this neuropathy (6).

In the mouse, the phenotypes of the global null *Fig4^plt/plt^* mutant and the *Fig4^Floxed/Floxed^*, *Syn*-Cre conditional null in neurons demonstrate the severe effect of PtdIns(3,5)*P*_2_ deficiency in neurons (1,3,12). Further, survival of *Fig4^plt/plt^* mice is rescued by the *NSE*-*Fig4* transgenic mice, which express *Fig4* specifically in neurons (12). Interestingly, among the different sub-population of neurons, DRG sensory neurons and autonomic ganglia are among the earliest affected neurons in the *Fig4^plt/plt^* mouse beginning by P1–P7, whereas motor neurons do not display typical pathological features related to PtdIns(3,5)*P*_2_ deficiency until 6 weeks of age (3,13). Whether *FIG4* has a cell autonomous role in motor neurons and Schwann cells and how loss of FIG4/PtdIns(3,5)*P*_2_-mediated functions in these cells contribute to CMT4J pathogenesis have not been clearly defined. To address this gap in our knowledge we generated conditional mutants that selectively lack *Fig4* in motor neurons or Schwann cells. Spinal cords of *Fig4*^*Floxed*/*plt*^, *HB9*-Cre and *Fig4*^*Floxed*/*plt*^, *Olig2*-Cre mice display motor neuron vacuolization, which is associated with axonal degeneration, hypomyelination and a decrease in the number of myelinated fibres in the quadriceps nerve. These findings suggest that *Fig4* has a cell autonomous role in motor neurons and that loss of *Fig4* in motor neurons contributes to the CMT4J neuropathy. In the motor neuron-specific knockout demyelinating features were not observed, supporting the hypothesis that demyelination is a consequence of specific loss of *Fig4* in Schwann cells. Consistent with this, we also report that *Fig4* conditional mutants in Schwann cells (*Fig4^Floxed/Floxed^*, *P0*-Cre and *Fig4^Floxed^*^/*plt*^, *P0*-Cre mice) exhibit accumulation of vesicles and organelles in the cytosol of myelinating cells indicative of a general trafficking impairment of the endolysosome axis, as well as enlargement of LE/LY and a block in autophagy. These cellular features are associated with impaired myelination during development, demyelination and defective regeneration, thus clearly demonstrating a primary involvement of Schwann cells in the pathogenesis of CMT4J neuropathy.

Demyelination in peripheral neuropathies can arise as a consequence of impaired endosomal trafficking and/or of a gain of toxic function. In addition to FIG4, several other proteins involved in the regulation of membrane trafficking are mutated in demyelinating CMT neuropathies and dys-regulation of endolysosomal trafficking may be a common mechanism at the basis of these disorders. These proteins regulate or are connected with PI metabolism, and include FRABIN/FGD4, SIMPLE, SH3TC2, MTMR2, MTMR5 and MTMR13 (1,35). For instance, mutated SIMPLE re-localizes from endosomal membranes to the cytosol where it exerts a dominant negative effect on the ESCRT protein complex (36,37). This in turn is thought to lead to defective ESCRT-mediated endosomal sorting and degradation of ErbB2/B3 receptors, loss of ERK signalling attenuation and demyelination.

Demyelination in peripheral neuropathies can also arise from activation of an integrated stress response (ISR) in Schwann cells. Several mutations in the myelin protein P0 responsible for demyelinating CMT1B cause misfolding of the protein, retention in the ER and UPR-mediated activation of ISR, which is maladaptive and leads to demyelination (38,39). Moreover, in the Tfam-KO Schwann cell mutant, mitochondria dysfunction causes ISR and demyelination (40). The *Fig4^Floxed/Floxed^*, *P0*-Cre and *Fig4^Floxed/plt^*, *P0*-Cre mutants that we generated with conditional ablation of *Fig4* in Schwann cells displayed demyelinating features such as myelin debris and degeneration, onion bulbs and decrease of NCV, thus recapitulating demyelination of the CMT4J neuropathy. Demyelination in these models can be the consequence of a general endolysosomal trafficking impairment and loss of LE/LY homeostasis. We indeed demonstrated that *Fig4*-deficient Schwann cells display a block in autophagy, which progressively leads to an accumulation of ubiquitinated substrates. As in lysosomal storage disorders, accumulation of macromolecules, cholesterol and defective calcium release from the LE/LY compartment may affect the functioning of several organelles such as Golgi, ER and mitochondria thus ultimately leading to demyelination (41).

### Endolysosomal trafficking and myelination

Myelin biogenesis relies on polarized trafficking and assembly of bulk of lipids and proteins (25). We exploited the mouse model with conditional ablation of *Fig4* in Schwann cells to investigate whether the resulting impairment of endolysosomal trafficking would influence myelin biogenesis during development. We indeed observed decreased myelin thickness and hypomyelination both *in vivo* and *in vitro* as a result of the *Fig4* deficiency in Schwann cells. During postnatal nerve development, the P0 myelin protein has been found to co-localize with the lysosomal cathepsin D in Schwann cells *in vivo*. Calcium-dependent lysosomal exocytosis has been suggested as a mechanism of P0 delivery to the plasma membrane during myelin biogenesis (27). We thought that hypomyelination in the conditionally deleted Schwann cells could result from the reduced delivery of P0 to the plasma membrane from the LE/LY compartment. However, we did not observe co-localization of P0 with LAMP1-positive LE/LY in either control or mutant Schwann cells at various stages of differentiation. We then hypothesized that the hypomyelination could result from a general impairment of trafficking occurring in *Fig4*-null Schwann cells. The observation that cholesterol supplementation does not rescue the hypomyelination *in vitro* supports this hypothesis. In normal conditions, cholesterol promotes the insertion of myelin P0 and other proteins into vesicles specifically destined for the developing myelin sheath. In *Fig4*-null Schwann cells, myelin proteins and/or cholesterol may be mis-trafficked and not properly delivered to the nascent myelin sheath causing hypomyelination.

Thus, we conclude that FIG4-mediated regulation of endolysosome trafficking in Schwann cells is important for generation of myelin during development, although the molecular mechanism remains to be clarified.

### Therapeutical perspectives for CMT4J and pathologies associated to FIG4 loss

FIG4 loss leads to decreased PtdIns(3,5)*P*_2_ levels and to the enlargement of LE/LY. Loss of LE/LY homeostasis and vacuolization in models of PtdIns(3,5)*P*_2_ deficiency may arise as a consequence of Ca^2+^ retention in the lysosome, which is the other Ca^2+^ buffering organelle in addition to the ER (41,42). Indeed, PtdIns(3,5)*P*_2_ activates the cation channels TPC1 (two pore channel), TPC2 and MCONL1/TRPML1 (Mucolipin 1, transient receptor potential cation channel subfamily M, member 1) localized at LE/LY compartments (43). An exciting and intriguing therapeutic strategy for CMT4J and other human disorders associated with FIG4 deficiency would be to increase the activity of these channels to restore LE/LY homeostasis and dynamics. Indeed, synthetic compounds have been recently identified as activators of the MCONL1/TRPML1 channels, which promote the efflux of Ca^2+^ from the lysosomal lumen into the cytosol and trigger lysosomal exocytosis (43). However, different cell types may respond differently to these treatments depending on the level of expression of these channels and the elicited electrophysiological response. Mouse models of cell-specific *Fig4* deficiency will therefore be useful for preclinical validation of these strategies.

## MATERIALS AND METHODS

### Ethics statement

All experiments involving animals were performed in accordance with Italian national regulations and covered by experimental protocols reviewed by local Institutional Animal Care and Use Committees (IACUC #525). Animals were generated and maintained in a SPF (specific pathogen free) Institutional facility.

### Mice

*Fig4^plt/plt^* (global *Fig4* deficiency) mice were maintained on CAST/Ei, C57BL/6J and 129 SV strain backgrounds. The generation of the *Fig4^Floxed^* allele has been already reported (12). The *Fig4^Floxed^* allele was detected by PCR with primers flanking exon 4 and the lox*P* sites. The forward primer, 5′-GAGGCAAGTACTCTACCAACTTAGC-3′ and reverse primer, 5′-CATGTGAACCTTGTTTCCCACACC-3′ detected fragments 558, 679 and 282 bp from wild type, *Fig4^Floxed^* and exon4-deleted alleles, respectively. *Fig4^Floxed/plt^ Olig2*-Cre mice were obtained by crossing *Fig4^plt^*^/+^ with wild-type mice carrying the *Olig2*-Cre transgene. The resulting *Fig4^plt^*^/+^, *Olig2*-Cre genotype was then crossed with *Fig4^Floxed/Floxed^* mice to obtain the *Fig4^Floxed/plt^*, *Olig2*-Cre conditional knock out. The same strategy was followed to generate *Fig4^Floxed/plt^*, *HB9*-Cre and *Fig4^Floxed/plt^*, *P0*-Cre. *Fig4^Floxed/Floxed^*, *P0*-Cre mice were generated by crossing *Fig4^Floxed/Floxed^* or *Fig4^Floxed/+^* with *Fig4^Floxed/+^*, *P0*-Cre mice. Genotyping was performed as already described (3,12,17). For all the experiments involving animals at least *n* = 4 animals per genotype of either sex were analysed. *Fig4^Floxed/Floxed^* mice have normal physiology and are indistinguishable from controls since they display normal nerve morphology including myelin thickness, and the absence of cell vacuolization in all cells analysed in both CNS and PNS also in aged mice (data not shown and as previously reported) (12). *Fig4^plt/+^* heterozygous mice are normal as no vacuolization has been observed in any tissue analysed and myelin thickness is normal in peripheral nerves, as previously reported (3,18).

### Morphological analysis

Semithin section and ultrastructural analysis of sciatic nerves was performed as previously reported (44). To perform morphometric analysis, digitalized images of fibre cross sections were obtained from corresponding levels of the sciatic nerves with a ×100objective and digital camera Leica DFC300F. Five images per animal were acquired and analysed with the Leica QWin software (Leica Microsystem). The g-ratio was determined by dividing the mean diameter of an axon (without myelin) by the mean diameter of the same axon including myelin. Statistical analysis was performed on the mean g-ratio values of the different nerves (animals) analysed per genotype. The quadriceps nerve, the motor branch of the femoral nerve, was dissected at the level of the inguinal ligament (45).

Sciatic nerve crush-lesion was performed as reported (46).

### Antibodies

For western blot analysis and immunohistochemistry the following antibodies were used: goat anti-ChAT (Millipore); rat anti-LAMP1 (Iowa Hybridoma bank); Guinea pig anti-P62 (Progen); rabbit anti-LC3 (Sigma); rat anti-MBP (Chemicon); rabbit anti-NF-H (Millipore); mouse anti-TUJ1 (Promega); mouse anti-tubulin (Sigma); rabbit anti-actin (Sigma); mouse anti-FIG4 (Neuromab); rabbit anti-S100 (Sigma), rabbit anti-NGF receptor p75 (Millipore); chicken anti-P0 (Millipore) and mouse anti-P0 antibodies (kindly provided by J.J. Archelos); rabbit anti-ubiquitin (Santa Cruz); rhodamine phalloidin (Molecular Probes).

Protein lysates were prepared using a lysis buffer containing 1% Triton X-100 (or NP40 1% for autophagy marker evaluation), 50 mm Tris buffer, pH 8.0, 150 mm NaCl, 10 mm NaF, 1 mm Na vanadate, Complete (Roche) protease inhibitors.

### Immunohistochemistry on sections

#### Spinal cord

Juvenile (P21) and adult (P120–210) mice were overdosed with ketamine/xylosine and transcardially perfused with ice-cold PBS and 4% PFA. Spinal cords were post-fixed in 4% PFA overnight, cryoprotected in 30% sucrose and frozen in OCT compound on dry ice. Lumbar spinal cords were sectioned at 30–40 µm at levels L3–L6 and stored in PBS. Free-floating immunohistochemistry was performed on 5–10 spinal cord sections per mouse. Sections were incubated overnight in primary antibody solution (Goat anti-ChAT, Millipore 1:100, 1.5% NDS in PBST-x) at 4°C on a rocker. After three washes in PBST-x sections were incubated in biotinylated donkey anti-goat secondary antibody in PBST-x (1:500, Jackson Immunoresearch) for 1 h. Followed by three washes in PBST-x, sections were incubated in ABC Vectashield kit solution (Vector, as per manufacturer's instructions) for 1 h. Antibody binding was visualized using diaminobenzidine kit (Vector Labs, as per manufacturer's instructions). Sections were mounted on glass slides, dehydrated in series of ethanol solutions and imaged using a Leica inverted bright field microscope.

#### Sciatic nerve

Immunofluorescence on cryosections was performed as described (47) and examined with confocal TCS SP5 laser-scanning confocal (Leica) or Olympus BX (Olympus Optical) fluorescent microscope, and Zeiss Axiovert S100 TV2 with Hamamatsu OrcaII-ER. For immunohistochemistry, sciatic nerves were removed and rapidly snap-frozen in liquid nitrogen, either unfixed or previously fixed in buffered 4% PFA.

### Cell culture

Isolated mouse Schwann cells were prepared from P2–P3 pups. Sciatic nerves were dissected, washed in L15 medium and then incubated with trypsin 0.25% plus collagenase 130 U/ml (type I, Worthington) in DMEM for 50 min at 37°C. After incubation, DMEM plus 10% serum was added to block dissociation and cells were plated on poly-lysine/laminin coated coverslips in defined medium, DF [DF contains 1 : 1 Hams F12/DMEM supplemented with 100 µg/ml glutamine, 0.03% bovine serum albumin (BSA), 100 µg/ml transferrin, 16 µg/ml putrescine, 38 ng/ml dexamethasone, 60 ng/ml progesterone, 400 ng/ml thyroxine (T4), 5 ng/ml insulin (low insulin) or 5 µg/ml (high insulin), 10 ng/ml triiodothyronine (T3), 160 ng/ml selenium and 100 U/ml each of penicillin/streptomycin], 3% serum and Ara C. Schwann cells were further purified using DF without serum.

Myelin-forming Schwann cell/DRG neuron co-cultures were established from embryonic Day 13.5 mouse embryos as previously described (48,49). Following NB (Neurobasal, B27 supplement, glucose, NGF) medium treatment for 7 days to allow neuritogenesis and Schwann cell migration, myelination was induced by treatment for 7–15 days with ascorbic acid (final concentration, 50 μg/ml) (Sigma). Cholesterol (dissolved in ethanol) was added every other day to C-media (MEM, 10% serum, NGF, glucose) plus ascorbic acid at 20 μg/ml final concentration. Analysis of autophagy on co-cultures was performed by starving Schwann cell/DRG explants in HBSS for 6 h and then by adding 100 nm bafilomycin A1 (LC laboratories) for 2 h to block LC3/p62 lysosomal degradation.

Analysis of proteasome-mediated degradation was performed by treating co-cultures using MG132 proteasome inhibitor at 25 µm final concentration for 6 h.

For analysis of myelination, immunohistochemistry on Schwann cell/DRG neuron co-cultures was performed as follows: cells were fixed for 15 min in 4% paraformaldehyde, permeabilized for 15 min in ice-cold methanol at −20°C, blocked for 20 min with 10% normal goat serum (Dako), 1% BSA (Sigma) and then incubated with primary antibody for 1 h. After extensive washing, the coverslips where incubated with the secondary antibody for 30 min, washed and mounted. For double immunostaining with anti-NF-H and anti- MBP antibody, the coverslips were blocked with 1% BSA, 10% NGS for 20 min on ice, and primary antibodies were incubated overnight at 4°C. For LAMP1 staining, cells were permeabilized using 0.1% saponin after fixation.

### Analysis of myelination

To quantify the amount of myelin, the number of MBP-positive segments in each explant/coverslip was assessed. As myelination is also a function of the amount of neurites/axons and of the Schwann cell number in the culture, the network of NF-H-positive filaments and the number of Schwann cells (DAPI) were also evaluated in each explant. Using a fluorescence microscope, at least 5–10 fields per cover were randomly acquired and MBP-positive myelinated fibres were counted per field. Average (among 5–10 fields) of MBP fibres was calculated per cover and statistical analysis was performed on different covers per condition/per experiment (SEM) in at least three different experiments. In the case of cholesterol treatment, the entire DRG was also reconstructed to evaluate the difference in the area occupied by myelinated segments in treated versus untreated explants. MBP-positive fibres and vacuolated organelles were acquired using TCS SP5 laser-scanning confocal (Leica) or Olympus BX (Olympus Optical) fluorescent microscope, and Zeiss Axiovert S100 TV2 with Hamamatsu OrcaII-ER. P0-LAMP1 co-localization analysis was performed using DeltaVision-Olympus IX70 with DeltaVision RT Deconvolution System.

### Imaging and statistical analysis

Micrographs were acquired using a digital camera (Leica F300), and figures were prepared using Adobe Photoshop, version 7.0 and 8.0 (Adobe Systems). Statistical analysis was performed using the Student's *t-*test; two tails, unequal variance and *α* = 0.005 were used. Error bars in the graphs represent SEM.

## SUPPLEMENTARY MATERIAL

Supplementary Material is available at *HMG* online.

*Conflict of Interest statement*. None declared.

## FUNDING

A.B. was supported by Telethon-Italy grant numbers GPP10007D, GGP12017; Association Française contre les Myopathies (AFM)-France grant number 16040/16922, and the ERA-Net for research programs on rare diseases (E-Rare JTC2011). S.C.P. was supported by Telethon-Italy grant numbers GGP10007B, GGP12024. Y.A.M. was supported by the Cellular and Molecular Biology Graduate Program Training Grant T32GM007315, 2011–2013 and the Training Program in Organogenesis T32HD007505, 2014–2015. M.H.M. and R.J.G. received support from National Institute of Health R01 GM24872 and R01 NS081281 and the Dr Miriam and Sheldon Adelson Foundation on Neurorepair and Rehabilitation to R.J.G. Funding to pay the Open Access publication charges for this article was provided by Telethon-Italy.

## Supplementary Material

Supplementary Data

## References

[1] Vaccari I., Dina G., Tronchere H., Kaufman E., Chicanne G., Cerri F., Wrabetz L., Payrastre B., Quattrini A., Weisman L.S. (2011). Genetic interaction between MTMR2 and FIG4 phospholipid phosphatases involved in Charcot-Marie-Tooth neuropathies. PLoS Genet..

[2] McCartney A.J., Zhang Y., Weisman L.S. (2014). Phosphatidylinositol 3,5-bisphosphate: low abundance, high significance. BioEssays.

[3] Chow C.Y., Zhang Y., Dowling J.J., Jin N., Adamska M., Shiga K., Szigeti K., Shy M.E., Li J., Zhang X. (2007). Mutation of FIG4 causes neurodegeneration in the pale tremor mouse and patients with CMT4J. Nature.

[4] Chow C.Y., Landers J.E., Bergren S.K., Sapp P.C., Grant A.E., Jones J.M., Everett L., Lenk G.M., McKenna-Yasek D.M., Weisman L.S. (2009). Deleterious variants of FIG4, a phosphoinositide phosphatase, in patients with ALS. Am. J. Hum. Genet..

[5] Campeau P.M., Lenk G.M., Lu J.T., Bae Y., Burrage L., Turnpenny P., Roman Corona-Rivera J., Morandi L., Mora M., Reutter H. (2013). Yunis-Varon syndrome is caused by mutations in FIG4, encoding a phosphoinositide phosphatase. Am. J. Hum. Genet..

[6] Nicholson G., Lenk G.M., Reddel S.W., Grant A.E., Towne C.F., Ferguson C.J., Simpson E., Scheuerle A., Yasick M., Hoffman S. (2011). Distinctive genetic and clinical features of CMT4J: a severe neuropathy caused by mutations in the PI(3,5)P phosphatase FIG4. Brain.

[7] Zhang X., Chow C.Y., Sahenk Z., Shy M.E., Meisler M.H., Li J. (2008). Mutation of FIG4 causes a rapidly progressive, asymmetric neuronal degeneration. Brain.

[8] Cottenie E., Menezes M.P., Rossor A.M., Morrow J.M., Yousry T.A., Dick D.J., Anderson J.R., Jaunmuktane Z., Brandner S., Blake J.C. (2013). Rapidly progressive asymmetrical weakness in Charcot-Marie-Tooth disease type 4J resembles chronic inflammatory demyelinating polyneuropathy. Neuromuscul. Disord..

[9] Nakajima J., Okamoto N., Shiraishi J., Nishimura G., Nakashima M., Tsurusaki Y., Saitsu H., Kawashima H., Matsumoto N., Miyake N. (2013). Novel FIG4 mutations in Yunis-Varon syndrome. J. Hum. Genet..

[10] Baulac S., Lenk G.M., Dufresnois B., Ouled Amar Bencheikh B., Couarch P., Renard J., Larson P.A., Ferguson C.J., Noe E., Poirier K. (2014). Role of the phosphoinositide phosphatase FIG4 gene in familial epilepsy with polymicrogyria. Neurology.

[11] Lenk G.M., Ferguson C.J., Chow C.Y., Jin N., Jones J.M., Grant A.E., Zolov S.N., Winters J.J., Giger R.J., Dowling J.J. (2011). Pathogenic mechanism of the FIG4 mutation responsible for Charcot-Marie-Tooth disease CMT4J. PLoS Genet..

[12] Ferguson C.J., Lenk G.M., Jones J.M., Grant A.E., Winters J.J., Dowling J.J., Giger R.J., Meisler M.H. (2012). Neuronal expression of Fig4 is both necessary and sufficient to prevent spongiform neurodegeneration. Hum. Mol. Genet..

[13] Katona I., Zhang X., Bai Y., Shy M.E., Guo J., Yan Q., Hatfield J., Kupsky W.J., Li J. (2011). Distinct pathogenic processes between Fig4-deficient motor and sensory neurons. Eur. J. Neurosci..

[14] Arber S., Han B., Mendelsohn M., Smith M., Jessell T.M., Sockanathan S. (1999). Requirement for the homeobox gene Hb9 in the consolidation of motor neuron identity. Neuron.

[15] Li H., Arber S., Jessell T.M., Edlund H. (1999). Selective agenesis of the dorsal pancreas in mice lacking homeobox gene Hlxb9. Nat. Genet..

[16] Yang X., Arber S., William C., Li L., Tanabe Y., Jessell T.M., Birchmeier C., Burden S.J. (2001). Patterning of muscle acetylcholine receptor gene expression in the absence of motor innervation. Neuron.

[17] Bolis A., Coviello S., Bussini S., Dina G., Pardini C., Previtali S.C., Malaguti M., Morana P., Del Carro U., Feltri M.L. (2005). Loss of Mtmr2 phosphatase in Schwann cells but not in motor neurons causes Charcot-Marie-Tooth type 4B1 neuropathy with myelin outfoldings. J. Neurosci..

[18] Yan Q., Guo J., Zhang X., Bai Y., Wang L., Li J. (2012). Trauma does not accelerate neuronal degeneration in Fig4 insufficient mice. J. Neurol. Sci..

[19] Lu Q.R., Yuk D., Alberta J.A., Zhu Z., Pawlitzky I., Chan J., McMahon A.P., Stiles C.D., Rowitch D.H. (2000). Sonic hedgehog--regulated oligodendrocyte lineage genes encoding bHLH proteins in the mammalian central nervous system. Neuron.

[20] Dimou L., Simon C., Kirchhoff F., Takebayashi H., Gotz M. (2008). Progeny of Olig2-expressing progenitors in the gray and white matter of the adult mouse cerebral cortex. J. Neurosci.

[21] Feltri M.L., D'Antonio M., Previtali S., Fasolini M., Messing A., Wrabetz L. (1999). P0-Cre transgenic mice for inactivation of adhesion molecules in Schwann cells. Ann. N. Y. Acad. Sci..

[22] Feltri M.L., D'Antonio M., Quattrini A., Numerato R., Arona M., Previtali S., Chiu S.Y., Messing A., Wrabetz L. (1999). A novel P0 glycoprotein transgene activates expression of lacZ in myelin-forming Schwann cells. Eur. J. Neurosci..

[23] Noseda R., Belin S., Piguet F., Vaccari I., Scarlino S., Brambilla P., Martinelli Boneschi F., Feltri M.L., Wrabetz L., Quattrini A. (2013). DDIT4/REDD1/RTP801 is a novel negative regulator of Schwann cell myelination. J. Neurosci..

[24] Anitei M., Pfeiffer S.E. (2006). Myelin biogenesis: sorting out protein trafficking. Curr. Biol..

[25] Simons M., Trotter J. (2007). Wrapping it up: the cell biology of myelination. Curr. Opin. Neurobiol..

[26] Lenk G.M., Meisler M.H. (2014). Mouse models of PI(3,5)P2 deficiency with impaired lysosome function. Methods Enzymol..

[27] Chen G., Zhang Z., Wei Z., Cheng Q., Li X., Li W., Duan S., Gu X. (2012). Lysosomal exocytosis in Schwann cells contributes to axon remyelination. Glia..

[28] Trajkovic K., Dhaunchak A.S., Goncalves J.T., Wenzel D., Schneider A., Bunt G., Nave K.A., Simons M. (2006). Neuron to glia signaling triggers myelin membrane exocytosis from endosomal storage sites. J. Cell Biol..

[29] Saher G., Quintes S., Mobius W., Wehr M.C., Kramer-Albers E.M., Brugger B., Nave K.A. (2009). Cholesterol regulates the endoplasmic reticulum exit of the major membrane protein P0 required for peripheral myelin compaction. J. Neurosci..

[30] Michell R.H., Heath V.L., Lemmon M.A., Dove S.K. (2006). Phosphatidylinositol 3,5-bisphosphate: metabolism and cellular functions. Trends Biochem. Sci..

[31] Michell R.H. (2013). Inositol lipids: from an archaeal origin to phosphatidylinositol 3,5-bisphosphate faults in human disease. FEBS J..

[32] Dove S.K., Dong K., Kobayashi T., Williams F.K., Michell R.H. (2009). Phosphatidylinositol 3,5-bisphosphate and Fab1p/PIKfyve underPPIn endo-lysosome function. Biochem. J..

[33] Ferguson C.J., Lenk G.M., Meisler M.H. (2009). Defective autophagy in neurons and astrocytes from mice deficient in PI(3,5)P2. Hum. Mol. Genet..

[34] Benbrook D.M., Long A. (2012). Integration of autophagy, proteasomal degradation, unfolded protein response and apoptosis. Exp. Oncol..

[35] Bucci C., Bakke O., Progida C. (2012). Charcot-Marie-Tooth disease and intracellular traffic. Prog. Neurobiol..

[36] Lee S.M., Chin L.S., Li L. (2012). Charcot-Marie-Tooth disease-linked protein SIMPLE functions with the ESCRT machinery in endosomal trafficking. J. Cell Biol..

[37] Lee S.M., Olzmann J.A., Chin L.S., Li L. (2011). Mutations associated with Charcot-Marie-Tooth disease cause SIMPLE protein mislocalization and degradation by the proteasome and aggresome-autophagy pathways. J. Cell Sci..

[38] D'Antonio M., Musner N., Scapin C., Ungaro D., Del Carro U., Ron D., Feltri M.L., Wrabetz L. (2013). Resetting translational homeostasis restores myelination in Charcot-Marie-Tooth disease type 1B mice. J. Exp. Med..

[39] Pennuto M., Tinelli E., Malaguti M., Del Carro U., D'Antonio M., Ron D., Quattrini A., Feltri M.L., Wrabetz L. (2008). Ablation of the UPR-mediator CHOP restores motor function and reduces demyelination in Charcot-Marie-Tooth 1B mice. Neuron.

[40] Viader A., Sasaki Y., Kim S., Strickland A., Workman C.S., Yang K., Gross R.W., Milbrandt J. (2013). Aberrant Schwann cell lipid metabolism linked to mitochondrial deficits leads to axon degeneration and neuropathy. Neuron.

[41] Platt F.M., Boland B., van der Spoel A.C. (2012). The cell biology of disease: lysosomal storage disorders: the cellular impact of lysosomal dysfunction. J. Cell Biol..

[42] Lloyd-Evans E., Platt F.M. (2011). Lysosomal Ca(2+) homeostasis: role in pathogenesis of lysosomal storage diseases. Cell Calcium.

[43] Samie M., Wang X., Zhang X., Goschka A., Li X., Cheng X., Gregg E., Azar M., Zhuo Y., Garrity A.G. (2013). A TRP channel in the lysosome regulates large particle phagocytosis via focal exocytosis. Dev. Cell.

[44] Wrabetz L., Feltri M.L., Quattrini A., Imperiale D., Previtali S., D'Antonio M., Martini R., Yin X., Trapp B.D., Zhou L. (2000). P(0) glycoprotein overexpression causes congenital hypomyelination of peripheral nerves. J. Cell Biol..

[45] Frei R., Motzing S., Kinkelin I., Schachner M., Koltzenburg M., Martini R. (1999). Loss of distal axons and sensory Merkel cells and features indicative of muscle denervation in hindlimbs of P0-deficient mice. J. Neurosci..

[46] Triolo D., Dina G., Lorenzetti I., Malaguti M., Morana P., Del Carro U., Comi G., Messing A., Quattrini A., Previtali S.C. (2006). Loss of glial fibrillary acidic protein (GFAP) impairs Schwann cell proliferation and delays nerve regeneration after damage. J. Cell Sci..

[47] Previtali S.C., Nodari A., Taveggia C., Pardini C., Dina G., Villa A., Wrabetz L., Quattrini A., Feltri M.L. (2003). Expression of laminin receptors in schwann cell differentiation: evidence for distinct roles. J. Neurosci..

[48] Taveggia C., Zanazzi G., Petrylak A., Yano H., Rosenbluth J., Einheber S., Xu X., Esper R.M., Loeb J.A., Shrager P. (2005). Neuregulin-1 type III determines the ensheathment fate of axons. Neuron.

[49] Bolis A., Coviello S., Visigalli I., Taveggia C., Bachi A., Chishti A.H., Hanada T., Quattrini A., Previtali S.C., Biffi A. (2009). Dlg1, Sec8, and Mtmr2 regulate membrane homeostasis in Schwann cell myelination. J. Neurosci..

