# Supplementary material for: Loss of Fig4 in both Schwann cells and motor neurons contributes to CMT4J neuropathy
Source: Hum Mol Genet. 2014 Sep 3;24(2):383–96. doi: 10.1093/hmg/ddu451 (PMC4275070; doi:10.1093/hmg/ddu451)
Supplement: Supplementary Data [file supp_24_2_383__index.html]

Loss of Fig4 in both Schwann cells and motor neurons contributes to CMT4J neuropathy — Loss of Fig4 in both Schwann cells and motor neurons contributes to CMT4J neuropathy — Supplementary Data 

# Loss of *Fig4* in both Schwann cells and motor neurons contributes to CMT4J neuropathy

## Supplementary Data

Supplementary Data

**Files in this Data Supplement:**

- Supplementary Data - Docx file
