# Supplementary material for: Loss of Fig4 in both Schwann cells and motor neurons contributes to CMT4J neuropathy
Source: Hum Mol Genet. 2014 Sep 3;24(2):383–96. doi: 10.1093/hmg/ddu451 (PMC4275070; doi:10.1093/hmg/ddu451)

**Supplementary Figure 1**. Conditional ablation of *Fig4* in motor neurons and the OL-lineage leads to vacuolization of spinal motor neurons. (**A**) Fig4 total protein levels are decreased in the spinal cords of *Fig4 ^Floxed^*^/^*^plt^, Olig2-*Cre mice at P21. Spinal cord homogenates of WT, *Fig4^plt^*^/^*^plt^,* *Fig4^Floxed/+^, Olig2*-Cre and *Fig4 ^Floxed^*^/^*^plt^, Olig2-*Cre mice were probed with anti Fig4 antibody. Neuronal marker TUJ1 was used as a loading control. N=2/genotype.

(**B**-**C’**) Choline acetylcholinetransferase (ChAT) positive neurons (arrows) in the ventral spinal cord containing cytoplasmic vacuoles in *Fig4 ^Floxed^*^/^*^plt^, Olig2-*Cre mice at P21 compared to control and at P120 (**D-E’’**). The arrowhead indicates large vacuoles forming exclusively in the ventral horn of the spinal cord of *Fig4 ^Floxed^*^/^*^plt^, Olig2-*Cre mice. Scale bars 500 μm, 200 and 50 μm (dashed box inserts). Controls are *Fig4^Floxed/+^, Olig2*-Cre and *Fig4 ^Floxed^*^/^*^plt^*.

**Supplementary Figure 2**. Autophagy is normal in *Fig4^Floxed/plt^,* *P0*-Cre hypomyelinated cultures at 7 days of ascorbic acid treatment. (**A**, **B**) LC3 II/I and p62 levels were normal in mutant cultures after starvation and Bafalomycin treatment as compared to controls. Each lane represents a pool of 12-15 DRG explants. Lysates from Hela cells starved or starved plus Bafalomycin treated were used as a positive control of LC3II/I and p62 elevation. (**C**) Lysates of Schwann cell/DRG explants treated or not treated using MG132 proteasome inhibitor to accumulate ubiquitinated substrates. No difference was observed between control and mutant cultures. Control genotypes are *Fig4^Floxed/+^*and *Fig4^plt/+^*.


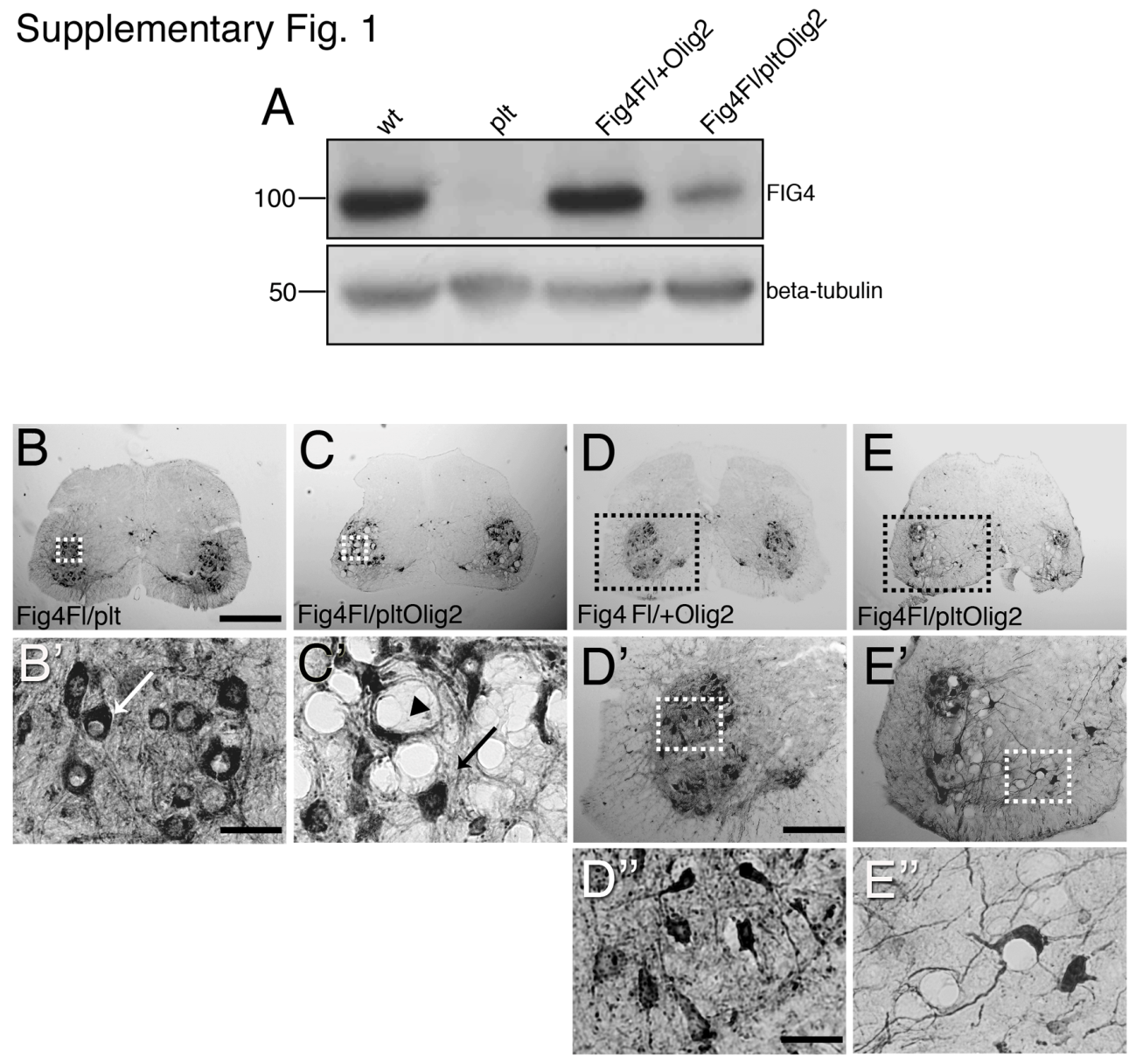


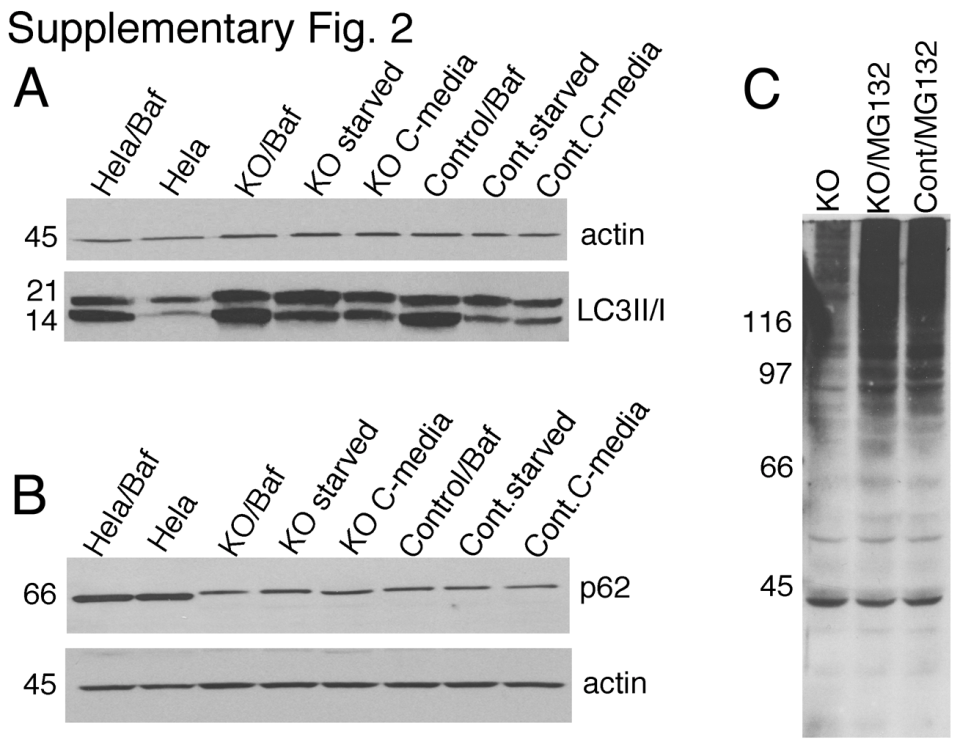

Supplement: Supplementary Data [file supp_ddu451_ddu451supp.docx]
